# Nodular osteochondrogenic activity in soft tissue surrounding osteoma in neurogenic para osteo-arthropathy: morphological and immunohistochemical study

**DOI:** 10.1186/1471-2474-5-46

**Published:** 2004-11-25

**Authors:** T Youssefian, R Sapena, R Carlier, C Bos, A Denormandie, P Denys, A Cormier, M Bandelier

**Affiliations:** 1 SYMPATHOS Laboratory, 67 boulevard du Général Martial Valin, 75015, Paris, France; 2 Medical Imaging department, Raymond Poincaré teaching hospital, APHP 104 Boulevard Raymond Poincaré, 92380, Garches, France; 3 Orthopedic deparment, Raymond Poincaré teaching hospital, APHP 104 Boulevard Raymond Poincaré, 92380, Garches, France; 4 Rehabilitation department, Raymond Poincaré teaching hospital, APHP 104 Boulevard Raymond Poincaré, 92380, Garches, France

## Abstract

**Background:**

Neurogenic Para-Osteo-Arthropathy (NPOA) occurs as a consequence of central nervous system injuries or some systemic conditions. They are characterized by bone formation around the main joints.

**Methods:**

In order to define some biological features of NPOAs, histological and immunohistological studies of the soft tissue surrounding osteoma and Ultrasound examination (US) of NPOA before the appearance of abnormal ossification on plain radiographs were performed.

**Results:**

We have observed a great number of ossifying areas scattered in soft tissues. US examination have also shown scattered ossifying areas at the early stage of ossification. A high osteogenic activity was detected in these tissues and all the stages of the endochondral process were observed. Mesenchymal cells undergo chondrocytic differentiation to further terminal maturation with hypertrophy, which sustains mineralization followed by endochondral ossification process.

**Conclusion:**

We suggest that periosteoma soft tissue reflect early stage of osteoma formation and could be a model to study the mechanism of osteoma formation and we propose a mechanism of the NPOA formation in which sympathetic dystony and altered mechanical loading induce changes which could be responsible for the cascade of cellular events leading to cartilage and bone formation.

## Background

Neurogenic Para Osteo-Arthropathies (NPOA) occurs in patients with brain or spinal cord injury, hemiplegias, various encephalopathies, tetanus [1] or neurological disregulation [2]. In this process, new bone named "osteoma" forms in extraskeletal areas which in normal condition do not ossify. NPOA were first described by Dejerine and Cellier [3] from observations of medullary wounded soldiers. They proposed the term NPOA, though other terms are used, such as: neurogenic osteoma, ossifying myositis in paraplegic, ectopic ossification, heterotopic ossification, etc. NPOAs have also been described as complications of many systemic diseases [4], acute pancreatitis, toxic syndromes and others [5].

The first clinical manifestations are local inflammatory signs, tumefaction and progressively limited range of motion of the involved joint region. Those appear between the second and tenth weeks after the onset of the pathological condition [6]. Despite anti-inflammatories treatment to prevent NPOA [7], excision of the newly formed bone called "osteoma", is the only known therapy.

As shown by radiographic and scintigraphic observations, heterotopic bone formation evolves from an early appearance of soft tissue densification and attenuation of the muscle signal to a mineral signal [8]. After 6 months, osteoma rarely increases in amount, but some further maturation occurs. As an assumption based on the fading of technetium fixation, the lesion is supposed to be mature after 1 to 1.5 years [9,10]. Hence, the process of NPOA formation seems to be frozen at the time of osteoma mineralization.

Very little is known about the pathophysiology of NPOA formation. Assuming such a freezing of the process of NPOA formation and an involvement of the periosteoma tissues in the reported relapses following surgery, we postulated that the periosteoma soft tissues could show some of the very early stages of the NPOA formation. We performed histological, histochemical and immunohistochemical studies of soft tissues dissected from the periphery of osteomas. We used samples of varying age lesions and searched for the main osteogenic and chondrogenic markers: alkaline phosphatase (ALP) activity, type I collagen and osteocalcin (OCN) for the bone [11-13], and type II collagen, sulfated and acid glycosaminoglycans, type X collagen and Vascular Endothelial Growth Factor (VEGF) for the cartilage [14]. In the light of our results, we propose a model of NPOA formation.

## Methods

### a)Specimen processing and histochemicals

The 28 specimens were obtained from 27 patients undergoing orthopedic surgery for osteoma excision. NPOA's were localized on: elbows (7), hips (18) and knees (3). The time from the neurologic insult ranged from 5 months to 216 months. The initial conditions were: 11 Brain Injuries (BI), 3 Spinal Cord Injury (SCI), 1 BI plus SCI, 4 strokes, and 9 patients sustained coma of various etiology (legionellose, anoxia, toxic condition, pneumonia, suicide attempt using neuroplegic).

**Figure 1:**
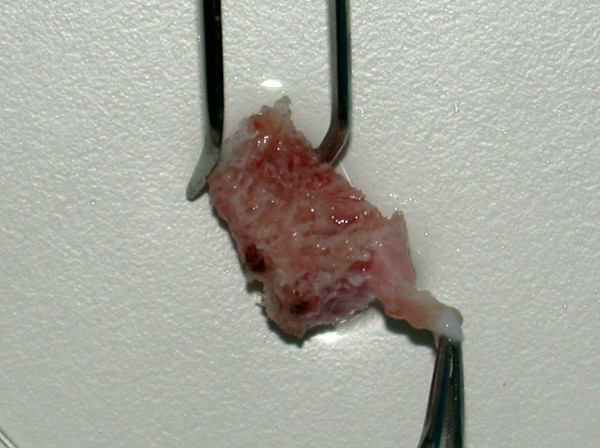
Dissection of part of the soft tissue surrounding a piece of osteoma.

Specimens obtained during the course of surgery, referred to in this paper as "osteoma", were immediately placed in sterile Gibco Hanks' balanced salts solution (Invitrogen, Cergy-Pontoise, France) at 4°C for transportation. The soft connective tissue was easily dissected off from the osteoma in order to exclude any part of the bony mass (Fig 1). The specimens were fixed in 4% paraformaldehyde in Phosphate Buffered Saline (PBS) with 0.5 M sucrose, frozen in isopentane in liquid nitrogen and stored at -86°C until embedding in OCT compound (Tissue-Tek, Sakuran Zoeterwoude, The Netherlands). Cryosectioning was performed on a Leica CM 3050 S cryostat (Leica Micro-Systems, Reil-Malmaison, France) at a thickness of 7 μm. Histochemical staining was performed according to standard protocols: Erlich's hematoxylin-eosin for general topographic staining, alcian blue pH1 for sulfated acid glycosaminoglycans, Von Kossa to show calcified areas, Oil red O to identify lipids, and Van Gieson Picro-Fuchsine for collagen distribution. The ALP-activity was demonstrated by using the Sigma procedure n°86 (Sigma Diagnostics, Saint Quentin Fallier, France). Slides were examined on a Leica DMR microscope (Leica Micro-Systems, Reuil-Malmaison, France), and pictures were recorded using a CCD colour camera with the Q Fluoro and Lida software systems (Leica Micro-Systems, Reil-Malmaison, France).

### b)Immunohistochemistry

The anti-human monoclonal mouse antibodies against type II collagen (NeoMarkers Inc., CA, USA), OCN (Interchim, Montlucon, France), anti-human polyclonal goat antibody against type I collagen (Santacruz Biotechnology, CA, USA), anti-rat polyclonal rabbit antibody against type X collagen (Calbiochem-Novabiochem, CA, USA) and anti-human polyclonal rabbit antibody against VEGF (Santacruz Biotechnology, CA, USA) were used at 1 mg/ml. Peroxidase-conjugated goat anti-mouse was purchased from Immunovision Technologies (CA, USA), peroxidase-conjugated goat anti-rabbit from Dako (Dako Corporation, CA, USA) and peroxidase-conjugated donkey anti-goat from Santacruz Biotechnology (CA, USA). They were used at a 1/20, 1/50 and 1/100 dilution, respectively. Frozen sections post-fixed with acetone were treated with hyaluronidase (5 mg/ml in Tris Buffered Saline) one hour at 37°C. For type X collagen immunostaining slides were treated first 15 minutes at 37°C with hyaluronidase (5 mg/ml in PBS), washed two times in PBS, then 15 minutes with chondroitinase (2 U/ml in PBS), and washed two times in PBS. Immunohistochemistry was performed using ABC method. Briefly, endogenous peroxydase activity was eliminated with 0.3% H_2_O_2 _until total clearing of oxygen bubbles. Non-specific protein binding was performed with 10% non-immune serum, same host as secondary antibody, in PBS with 1% Bovin Serum Albumin (Sigma, Saint Quentin Fallavier, France). Sections were then incubated with primary antibodies for 1 hour at room temperature, or 24 hours at 4°C with anti-type I collagen. Excess antibody was removed by washing the sections with PBS. Sections were incubated 1 hour with horseradish peroxydase-labeled secondary antibody diluted in PBS. 3-3'diaminobenzidine (DAB) solution (Dako Corporation, CA, USA) was then added in order to obtain staining. Sections were counter-stained with hematoxylin-eosin or nuclear red/eosin, dehydrated, and mounted with Mountex medium (Microm, France). Controls were systematically performed omitting the primary antibody. Slides were examined by light microscopy using a Leica DMR microscope (Leica Micro-Systems, Reil-Malmaison, France).

### c)Ultrasound (US) Examination

Most of the patients were referred to Raymond Poincaré teaching hospital, at the time of surgery. US examination was performed when NPOA was clinically suspected in five patients whose rehabilitation has begun. Five hips were explored by US in two BI and three SCI. Linear 8 to 15 MHz and sectorial 4 MHz transducers (Sequoia Acuson-Siemens Erlangen) were used. A sectorial low frequency transducer was used because in the hip area NPOA can be very large and very deep especially in the gluteus area compared to the subcutaneous plane. US examination was combined with color and energy Doppler. In all cases a plain film was obtained the same day as the US examination.

## Results

### a)Histological and immunohistological studies

**Figure 2:**
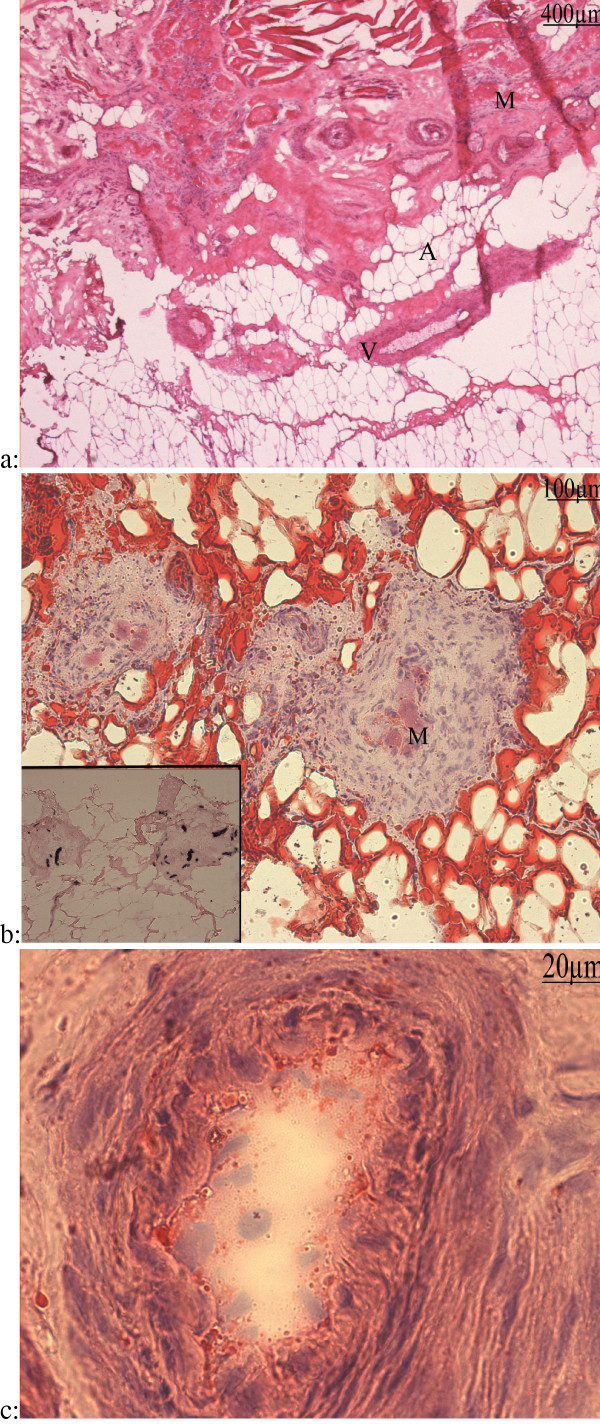
**a: Frozen section of soft tissue from a 72 months hip NPOA**: Hematoxylin eosin (h&e): Thrombotic vessels (V), more or less advanced vacuolization/degeneration of muscular fibers (M), and adipous tissue (A). **b: **Oil Red-O staining : Degenerated muscle fibers (M) stained by eosin were embedded in a strongly hyperplastic perimysium. This structure was itself located inside an adipous tissue whose appearance and compartmentalization by endomysium-like sheets of cell layers suggests a muscular origin. Inset: ALP activity: the same area showed an intense ALP activity in some cells in hyperplastic perimysium. **c: **Hematoxylin eosin (h&e) staining showed hyperplastic intima and media.

Non mineralized connective tissues from the periphery of the osteoma were examined by light microscopy on Erlich's hematoxylin-eosin-stained sections. Several kinds of tissues appeared on the slides so the diversity of these figures deserves a systematic analysis which will be completed in the next sections. Briefly, the ground basis of our preparations was a more or less fibro-cellular connective tissue displaying sometimes edema and/or necrosis. Suffering and degenerating tissues with vacuolized myofibers, thrombotic vessels and adipose tissue were often observed (Fig 2a). Muscular tissue underwent degeneration as shown by the vacuolisation or the disappearance of the internal eosinophily. Oil red-O stained adipocytes in the vicinity of some degenerating muscle with hyperplastic endomysium and perimysium (Fig 2b). In these regions, ALP activity was detected in the endomysium and perimysium cells of degenerated muscles (Fig 2b, inset).

**Figure 3:**
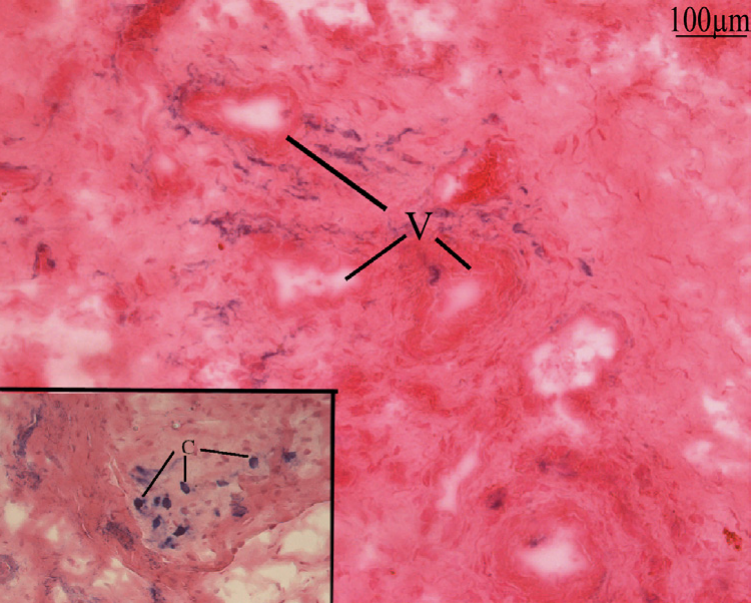
**Frozen sections of soft tissue of an 8 months hip NPOA**: Blue Alkaline Phosphatase (ALP) activity counter stained with nuclear red-eosin: perivascular cells show high ALP activity near by vessels (V) and some of these seems to migrate from these areas. Inset: Cluster and isolated rounded cells (C) with high ALP showed a more advanced stage of differentiation.

Morphologically normal vessels were rarely observed and winding of the vasculature was an almost constant finding. Many of the vessels were thrombotic, sometimes with hyperplastic intimae or media (Fig 2c). Some perivascular cells showed ALP activity and seemed to migrate out from these proliferating zones (Fig 3). Clustered or isolated round cells with high ALP activity were also observed embedded in a high amount of collagen matrix (Fig 3, inset).

**Figure 4:**
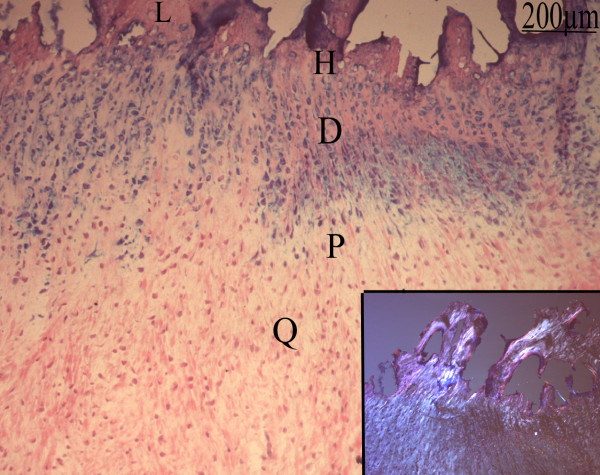
**Frozen section of soft tissue of an 8 months hip NPOA**: Blue Alkaline Phosphatase (ALP) activity counter stained with nuclear red-eosin: The successive stage of chondrocytes differentiation were observed: quiescent (Q), proliferative (P), prehypertrophic (D), hypertrophic (H) and mineralization zones. Inset: Lamellar bone deposition (L) is visualized with polarized light.

These findings point to a chondrogenic or osteogenic differentiation of formerly undifferentiated mesenchymal cells from the stroma and the vessel walls. More advanced stages of cartilaginous differentiation were frequently observed in avascular areas with varying degrees of chondrocyte maturation. Morphologically recognizable columns of chondrocyte-like cells presenting a high ALP activity were observed. Moreover, we could observe all the stages of progressive chondrocyte differentiation from quiescence to chondrocyte hypertrophy/matrix mineralization and endochondral ossification (Fig 4).

**Figure 5:**
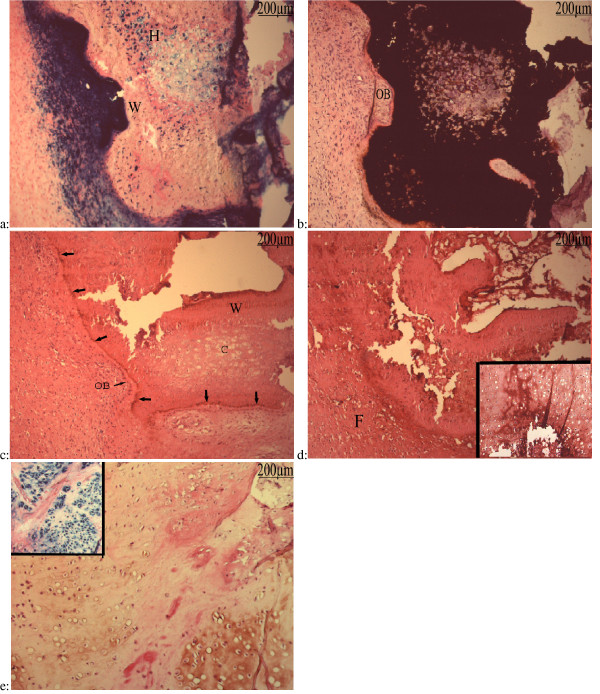
**Frozen sections of soft tissue of a 5 months elbow NPOA**: **a: **Blue Alkaline Phosphatase (ALP) activity counter stained by nuclear red-eosin: A very strong ALP activity in multilayered cells surrounded a peripherically mineralized area. This area was made of woven bone (W) deposed on an hypertrophic cartilage (H) centered by prehypertrophic chondrocytes. Hypertrophic and prehypertrophic chondrocytes in the non-mineralized matrix displayed ALP activity. **b: **Vonkossa staining of a next section counter stained by h&e: Von Kossa stain was a negative image of the ALP activity. Osteoid matrix was deposed upon the calcified cartilage matrix and underwent mineralization. Palissade-arranged cells lined this osteoid and displayed morphological image of osteoblast-like cells (OB). **c: **Oscteocalcin immunolabelling of a next section of the same specimen counter stained by h&e: Osteocalcin immunoreactivity in the eosinophilic osteoid at the border of the mineralized woven bone and sligtly on osteoblast-like cells (OB) lining this zone. Slight labelling was also present in the woven bone (W), but not in the cartilage (C) areas. **d: **Type X collagen immunolabelling of a next section of the same specimen counter stained by h&e: Type X collagen immunoreactivity was found in the mineralized cartilage area up to the woven bone. It also streched over most of the fibrous tissue (F) surrounding the mineralized area. Inset: It was also present in hypertrophic chondrocytes and their matrix. **e: **Type II collagen immunolabelling of this specimen conter stain by h&e: The matrix of the columnar cartilage was immunolabelled with type II collagen antibody. Remnants of eosinophilic degenerated muscle fibers were interspersed among the cartilage. Inset: The type II colllagen immunoreactive areas displayed an heavy ALP activity.

A high ALP activity was also found in cells surrounding the cartilage areas undergoing mineralization and embedded in a slight envelope of woven bone with ALP positive cells (Fig 5a). On the other hand, ALP activity of hypertrophic chondrocytes was progressively lost as mineralization occurred, thus Von Kossa staining seemed to be a negative image of ALP activity (Fig 5b).

Bands of eosinophilic material stained partially by Von Kossa underlined some borders of the mineralized cartilage. These osteoid-like bands were lined by cells which morphologically appeared to be osteoblast-like cells.

To confirm the osteoblastic nature of these cells, immunolabelling for OCN was performed (Fig 5c). In front of the osteoblastic-like cells a consistent matricial OCN immunoreactivity was evident onto the eosinophilic matrix underlining the mineralized cartilage. We have also observed deposition of OCN into woven bone formed adjacent to cartilage.

In order to document the collagenic composition of these tissues and confirm their osseous or cartilaginous nature, immunolabelling for type I, II and X collagens were performed.

Conspicuous immunolabelling for type X collagen was observed in most of the hypertrophic chondrocytes and in their matrix. The immunoreactivity became more intense nearby the mineralization front. In addition the matrix of the fibrous and non cartilage-like tissue around some mineralized areas was unexpectedly labelled (Fig 5d). Control samples in which primary antibody incubation was omitted were clearly negative (data not shown).

Distribution of type II collagen was limited to the matrix of non hypertrophic and prehypertrophic chondrocytes with high ALP activity (Fig 5e). Nevertheless some type II collagen immunoreactivity could sometimes be detected in the hypertrophic areas.

**Figure 6:**
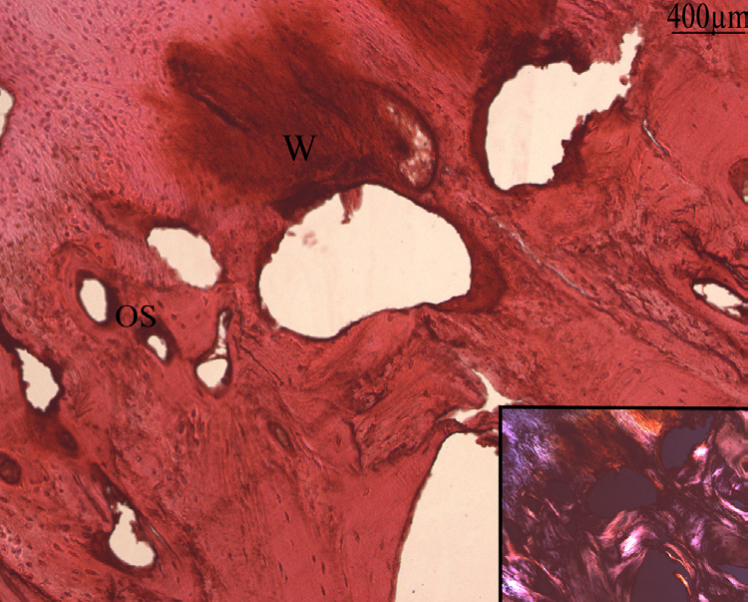
**Frozen section of soft tissue of a 24 months hip NPOA**: Type I collagen immunolabelling counter stained by h&e: Type I collagen was the main constituant of the matrix in the primary osteons (OS) and non organized woven bone (W). Inset: lamellar bone deposition observed by polarized light

Bone deposition was frequently observed by polarized light and confirmed by type I collagen immunostaining (Fig 6). Type I collagen was located in the osteoblast-containing matrix which formed and lined up along spicules of calcified cartilage. Osteocytes were trapped in the lamellar and woven bone with type I collagen immunoreactivity.

### b)Serial sections

In order to determine the chronology of events at work in the described endochondral ossification, we performed serial cryosectioning of samples in which a cortical bone followed soft tissue. These samples seem to be appropriate to have all stages of osteogenesis

**Figure 7:**
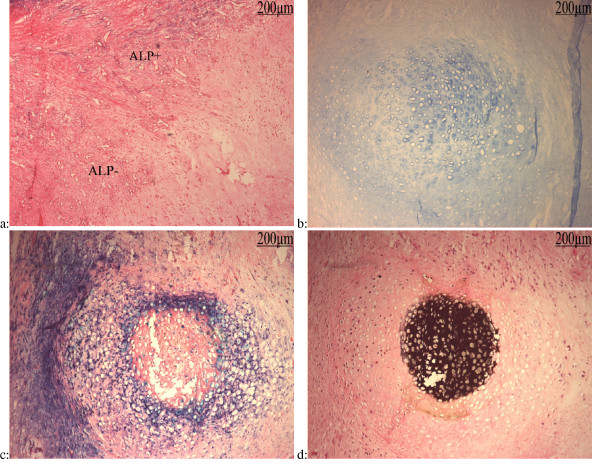
**Serial sections of soft tissue of 24 months hip NPOA**: **a: **ALP activity counter stained with nuclear red-eosin: Slide 118; Important angiogenesis encircles avascular areas. Many of these vessels express ALP activity (ALP+). **b: **Alcian bleu pH = 1 staining: Slide 70: chondroitin sulfate accumulation in cartilage. **c: **ALP activity counter stained with nuclear red-eosin: Slide 71. **d: **Von Kossa staining countre stained with h&e: Slide 65.

One of the specimens appeared to contain an aponeurotic tissue which showed signs of bursitis. In a highly cellular tissue we observed a high angiogenic activity. Bundles of vessels surrounded amorphous and avascular zones (Fig 7a). Some of these vessels expressed slight ALP activity which became more and more intense in the vicinity of the acellular areas. Then the avascular areas were replaced by nodules of cartilage with prehypertrophic and hypertrophic chondrocytes. These areas were stained by alcian blue pH1, showing chondroitin sulfate accumulation in their matrix (Fig 7b).

**Figure 8:**
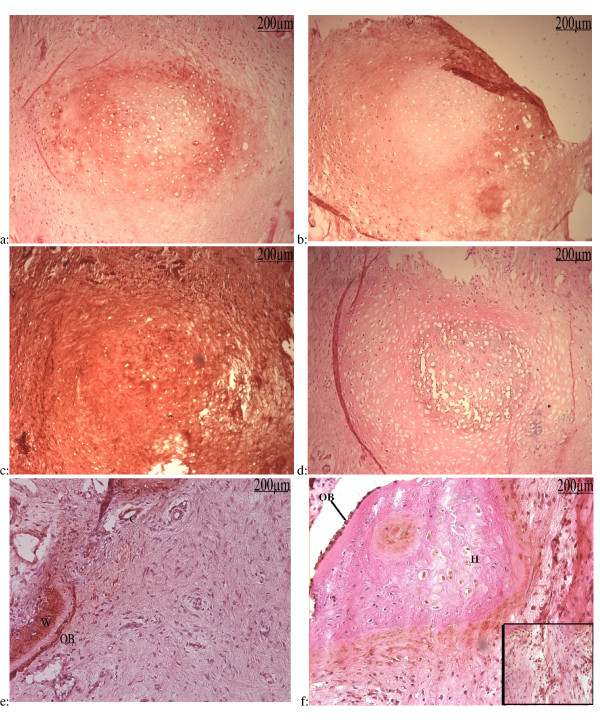
**Immunological study of serial sections**: **a: **Type II Collagen immunolabellingcountre stained with h&e: Slide 69: Type II collagen had the same pattern of ALP activity of nodule showing prehypertrophic chondrocytes in nonmineralized zone. **b: **Type I collagen Immunolabelling countre stained with h&e: Slide 72; Type I collagen expression encercled the mineralized nodule. **c: **Type X collagen immunolabelling countre stained with h&e:Slide 73: Type X collagen was expressed by most of cells and distributed in their matrix. **d: **OCN Immunolabelling countre stained with h&e: Slide 64: OCN was expressed by hypertrophic chondrocytes. **e: **OCN immunolabelling counter stained with h&e: Activated osteoblasts (OB) and woven bone (W) are strongly labelled. Some capillaries (C) near by these areas express osteocaline too. **f: **VEGF immunolabelling counter stained with h&e: VEGF was expressed by some hypertrophic chondrocytes (H). Activated and non activated osteoblasts-like cells (OB) lining the cartilage express also VEGF. Inset: Clustered and isolated cells in the matrix, surrounding hypertrophic chondrocytes, which could be destinated to capillary or osteoblast formation, are also labelled by VEGF.

At this stage a strong ALP activity was observed in the cells surrounding the cartilage zone as well as in non mineralized hypertrophic areas (Fig 7c). Finally Von Kossa staining revealed the matrix mineralization (Fig 7d). Immunolabelling of these sections with type II collagen antibody demonstrated a circle of prehypertrophic chondrocytes (Fig 8a). The matrix of the fibrous tissue outside this mineralized nodule was immunoreactive to type I collagen antibody (Fig 8b). Type X collagen recovered the nodule of hypertrophic chondrocytes and the rest of this section showing a high osteogenic activity (Fig 8c). OCN immunolabelling revealed exactly the same zone stained by Von Kossa showing deposition of OCN on mineralized zone (Fig 8d). As previously described OCN was detected in the osteoblast-like cells lining the newly lay down osteoid as well as in the newly formed woven bone and on areas of membranous bone formation (Fig 8e). OCN was also observed in some cells around the vessels near by the areas of osteogenesis.

To further confirm the process of endochondral ossification, we decided to search for VEGF expression. Immunolabelling of these tissues with VEGF monoclonal antibody, showed a labelling of the hypertrophic chondrocytes as well as an intense labelling of activated osteoblats lining the osteoid. Some clusters of rounded cells also expressed VEGF in the fibrous part of these preparations (Fig 8f).

### c)Ultrasound examination and digital radiographs of suspected NPOA

**Figure 9:**
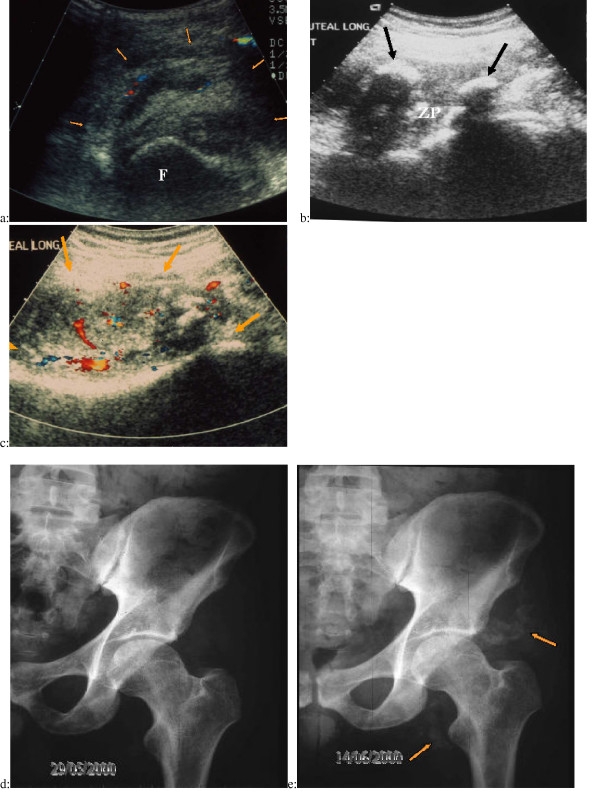
**Ultrasound and color Doppler examination and digital radiographs of suspected NPOA a: **Axial US view combined with color Doppler of the anterior side of the left hip in a paraplegic patient presenting acute limitation and inflammation of this joint. The striation of the psoas iliaque muscle, normally detectable at the anterior part of the hip joint with US examination, has disappeared. A relatively well defined mass (orange arrows) is detectable at the anterior part of the left femoral head (F). This mass is very heterogeneous with mixed hypo and hyper echoic areas. Color Doppler enables visualization of vessels in the mass (red and blue Doppler signals). A mass effect is visible on the femoral vessels (top right of the view). **b: **Same patient, one week later, axial US view of the posterior side of the left hip. The classical zone phenomenon (ZP) is detectable with a central hypoechoic area surrounded by hyper echoic nodules with posterior attenuation(black arrows). **c: **Axial US examination at the same day combined with color Doppler view of the posterior side of the left hip. A posterior mass (orange arrows) is also visible in the gluteal muscles, very heterogeneous with mixed hypo and hyper echoic areas. Color Doppler reveals large vessels in the mass (red and blue Doppler signals). **d: **Plain radiographs of the left hip obtained the same day as first US examination: Any sign of ossification is visible while a well defined mass is detected by US examination. **e: **Plain radiographs of the left hip obtained two weeks after: Early anterior and posterior NPOA ossification is only slightly visible two weeks (Orange arrows) after the initial clinical signs whereas the US examination was initially positive.

US examination showed a huge focal disorganization of the muscles in the area of the suspected NPOA. Normal longitudinal muscular striation disappeared and was replaced by a relatively well defined mass with a very heterogeneous echostructure. The masses ranged from six to eleven centimeters of long axis. No scattered ossified areas were detected by US at this stage. Hypervascularization was detected on Doppler examination inside and outside the NPOA tumors (Fig 9a).

Classical zone phenomenon previously described in the literature [15,16] was visible with a central hypo echoic area surrounded by small (less than one centimeter) hyper echoic nodules with posterior attenuation (Fig 9b). At this stage color Doppler examination showed increasing angiogenesis with the appearance of large vessels in the tumor mass (Fig 9c).

The zone phenomenon became visible on the second US examination performed one week later (Fig 9d).

The opacity of the early ossification became slightly visible on plain films only two weeks after the US detection of zone phenomenon (Fig 9e).

## Discussion

NPOA pathogenesis is still poorly understood, and the exact environmental and humoral conditions underlying the ossifying process are not clear. In this study we postulated that periosteoma soft tissues display interrupted early stages of osteoma formation, which could help us to understand the chronology as well as the mechanism of osteoma formation. Thus, histological and immunohistological experiments were performed on 28 specimens. Moreover, ultrasound examination of suspected NPOA tumor on five patients permitted to follow osteoma formation in the early stages before ossification.

Histological studies have shown varying amount of muscle and connective tissue degeneration in which some areas underwent reorganization. Islands of cartilage, woven bone, and mature lamellar bone were a constant finding in most of specimens, whatever the estimated age of the studied lesion. The spatial organization of chondrocytes was reminiscent of the epiphyseal growth plate or of the fracture callus organization [17]. In the developmental pathway leading to skeletogenesis, undifferentiated mesenchymal cells pass sequentially through at least four differentiation stages: committed mesenchymal cells which produce type I collagen and possibly basal level of type II collagen, quiescent chondrocytes, then proliferating chondrocytes characterized by the synthesis of a large amount of type II collagen and sulfated proteoglycans, and ultimately hypertrophic chondrocytes characterized by the synthesis of type X collagen. Then these hypertrophic chondrocytes allow the mineralization of the matrix elaborated and induce vascular invasion by releasing VEGF [18,19]. Studies of our specimens showed the same sequence of events. These results suggest that endochondral osteogenesis is the major pathway in the NPOA bone formation. Nevertheless in view of some features suggesting bone deposition without any cartilage scaffold we cannot exclude the occurrence of membranous bone formation.

Some sections showed a high expression of type X collagen in the hypertrophic chondrocytes areas, and unexpectedly in non differentiated mesenchymal tissue. As this labelling did not occur in other fibrocellular areas it was unlikely to be produced by a binding of the antibody to some other matricial component of the extra cellular matrix. It was shown, that type X collagen is not only associated with chondrocyte proliferation and hypertrophy, but also with resting chondrocytes, cells at the border of the perichondrium and resting cartilage of the fetal femoral head [20].

The finding of still degenerating muscular fibers and early chondro-osteogenesis accompanied by heavy ALP activity in large parts of the soft tissues in old lesions (till 8 years) is singular. Except when serial sectioning was performed the studied specimens were carefully dissected from the osteoma during the surgery or at the fixation time. Therefore the extra-osteoma localization of these tissues can without any doubt be assumed. In one study [21] "recent POA" was described as a kind of fibrocellular tissue including vascular stasis, overabundance of micro vessels, myolysis, edematous fibrocellular tissue, with chondrogenesis, osteogenesis and lamellar bone apposition on mineralized structures. This description of "recent POA" is perfectly in agreement with our description of the periosteoma tissues. However, we found each of these elements notwithstanding the advanced age of some lesions. Most of our specimens contained clusters of ALP positive cells in the undifferentiated fibrous connective tissue, suggesting the presence of preosteogenic cells. This fact and the presence of cartilage and bone at varying stages of maturity are indicative of a persistent chondro-osteogenic activity in these tissues. This point sounds of interest as regards to contingencies of heterotopic bone formation relapse following surgical excision. The occurrence of relapses was reported to correlate neither to the classical criteria of osteoma maturation nor to the amount of heterotopic bone left after excision nor to the age of the lesion [22-24]. The occurrence of relapses could be linked to the activation of the still process.

It was claimed that osteoma develops in the periphery of a muscle in which some myofibers undergo degeneration [25,26], and that osteoma involves the muscles. However the endochondral process of bone formation described here is in agreement with the results of various bone induction experiments in muscle [27-30]. Moreover, US examination of suspected NPOA tumor at the early stage showed huge focal disorganisation of muscle surrounding the hip joint and disappearance of normal longitudinal muscular striation of the psoas iliaque muscle replaced by masses with heterogeneous echostructure. This finding argues in favour of an intramuscular beginning of the process. We could not set up the place and part of the muscle degeneration process in the heterotopic bone formation. Though the process described here resembles by some aspects the Fibrodysplasia Ossificans Progressiva (FOP) where endochondral ossification was demonstrated in muscle and adjacent connective tissue [31,32]. These reports combined with our finding of endomysium-perimysium hyperplasia in the degenerating muscles with ALP activity and US result, could suggest a role for the muscular tissue and especially mesenchymal cells from endomysium and perimysium in the setting of the heterotopic bone formation process. Vascular disorders, such as vascular disruption or compression [33] and venous stasis together with a cascade of inflammatory reactions including release of enzymes from necrotic tissues and α-adrenergic mediated vasoconstriction, lead to the formation of hypoxic zones beneath the nervous injury.

On the other hand it has been shown that hypoxia promotes chondrocyte differentiation [34,35]. In our specimens, high amount of thrombotic vessels were indicative of the hypoxic status of these tissues. Color Doppler examination of tumor during the first clinical signs of NPOA, showed an avascular area in which some vessels appeared. These signals became more intense one week later. This tends to confirm hypoxic status of the initial lesion. This suggests that a timely defined hypoxic condition in tissue induces chondrocytes differentiation.

The volume of the tumor is acquired during the very early stages of NPOA. After tumefaction there is no real change in the tumor size [2]. Moreover chondrocytes in the hypertrophic stage increase volume 10 times [36]. So the chondrocyte hypertrophy could be the cause of the tumefaction and so determines the final size of the tumor. Then, the related vascular sign occurs and color Doppler showed the first sign of increasing angiogenesis.

On tissue section, VEGF immunolabelling revealed a more intense expression by osteoblasts and osteoblast-like cells, in addition to its expected expression by hypertrophic chondrocytes. VEGF induces neoangiogenesis and then endochondral ossification occurs. The restoration of normoxic conditions promote the onset of lamellar ossification and hamper any other *de novo *cartilaginous differentiation. US examination of the same NPOA tumor one week later showed scattered ossifying nodules. This nodular activity is in accordance with our histological finding in periosteoma soft tissue. Extense of the hypoxic area determines the size of each nodule. Areas with important hypoxia induce larger cartilage zones which could join together after ossification but small nodules remain scattered in periosteoma soft tissue. These results confirm that, periosteoma soft tissue has the same pattern of early stage of osteoma ossification, and could be a model of ossification for further studies.

Moreover, NPOA occurs in neurologically deficient patients with altered mechanical loading. Mechanical loading is of pivotal importance to the development, function and repair of all tissues in the musculoskeletal system. In nonfunctional joints, as it is the case of these patients, the absence or reduction of intermittent hydrostatic pressure in the articular cartilage could permit cartilage degeneration and the progressive advance of the ossification These mechanical influence could indeed shed light on the finding that osteomas only occur near the main joints.[37]

Moreover, Carter and associates have also shown that intermittently applied shear stresses (or strain energy) promote endochondral ossification and that intermittently applied hydrostatic compression inhibits or prevents cartilage degeneration and ossification. Thus, the imbalance of these forces among these patients can promote endochondral ossification of the cartilage nodules in areas of high shear (deviatoric) stresses[38].

Urist demonstrated that the induced endochondral bone is resorbed once the inductive agent has disappeared [30]. We have not seen many osteoclasts nor other multinucleated cells in ours preparations, and the literature does not report on NPOA regression except in people under 15 years of age [39]. Therefore it would be interesting to study the regulation of osteoclasts and the remodelling in such model.

We therefore propose a model of the NPOA lesion formation. The sympathetic hyperactivity causes major changes in the peripheral vascular dynamics. As related some of these changes end in vascular stasis and/or thrombosis [2]. Edema follows on in the connective tissue which sustains some amount of necrosis. The trauma, subsequent neurological conditions and perhaps systemic factors [40,41] induce major changes in these tissues. Regenerating celles under low oxygen pressure/high dilatational hydrostatic forces [34,42,43] transmogrify themselves into chondrogenic cells. Then cartilage differentiation gets moving on until hypertrophy of the chondrocytes and cartilage matrix mineralization. The cell hyperplasia and hypertrophy of the chondrocytes could account for the solid swelling clinically noticed soon after the onset of the clinical signs of NPOA. Like they did in the developing limb, some competent cells lining the cartilage rudiments undergo the osteoblastic differentiation and lay down osteoid on the cartilage. Concomitantly the cartilage hypertrophy induces angiogenesis, osteoid deposition, and some extent of cartilage resorption. Remodelling of the mineralized cartilage and woven bone occurs. The osteoma could then control the process and inhibit any further bone formation.

Some questions remain which deserve further studies. Why do NPOAs form only around the main joints? Why are they not resorbed as does any intramuscularly implanted bone graft [44]. What freezes the osteoma bone growth and the process of bone formation? Studies are ongoing in order to find some clues about the regulation of this heterotopic bone formation.

## Conclusion

In conclusion, our results demonstrate that periosteoma soft tissues are a replica of the early stages of osteoma formation, and could be used as a model for NPOA formation. We propose also a mechanism for osteoma formation in which hypoxia is a major cause of nodular osteoinduction and chondrocyte differentiation. Combination of hypoxia and applied shear stresses induce endochodral ossification. Finally our results indicate implication of different types of mesenchymal cells in NPOA formation but US examination support specially muscular origin hypothesis.

## Pre-publication history

The pre-publication history for this paper can be accessed here:


